# Development of a quadruplex PCR amplicon next generation sequencing assay for detection and differentiation of *Bartonella* spp.

**DOI:** 10.3389/fmicb.2023.1243471

**Published:** 2023-12-07

**Authors:** Ying Bai, Lynn M. Osikowicz, Andrias Hojgaard, Rebecca J. Eisen

**Affiliations:** Bacterial Disease Branch, Division of Vector-Borne Diseases, Centers for Disease Control and Prevention, Fort Collins, CO, United States

**Keywords:** assay development, quadruplex, next-generation sequencing (NGS), *Bartonella* spp., differentiation

## Abstract

The genus *Bartonella* includes a group of species that are associated with a wide range of mammalian species, including human. It is challenging to detect all *Bartonella* species using a single molecular target due to its high genetic diversity. To solve this issue, we developed a quadruplex PCR amplicon sequencing assay using next-generation sequencing (NGS) technology for the detection and differentiation of *Bartonella* species. Our objective was to obtain the specific sequences of a minimum of two of the four target genes as confirmation of the identity of a particular *Bartonella* species using the assay. Four pairs of primers targeting specific regions on *gltA, groEL, rpoB*, and *ssrA* were evaluated for their capability of differentiating *Bartonella* species individually and collectively by performing singular PCR amplicon sequencing and quadruplex PCR amplicon sequencing. Using the quadruplex PCR amplicon sequencing, 24 *Bartonella* reference species were tested, all of which were successfully differentiated by at least two targets. *Bartonella s*pecies were accurately identified from the artificially mixed DNA templates developed to simulate coinfections. The limit of detection was determined to be 1 fg based on testing a series of 10-fold dilutions of DNA from the *Bartonella* species. Testing of high DNA concentrations of 19 non-*Bartonella* species showed high specificity with none of the non-*Bartonella* species misclassified as *Bartonella*. Finally, the assay was evaluated by testing DNA extracts from field-collected body lice (*Pediculus humanus humanus*) and Norway rats (*Rattus norvegicus*): *Bartonella quintana* was detected and confirmed by three targets in the lice and *Bartonella tribocorum* was detected and confirmed by two targets in the rats. These results demonstrated that *Bartonella* species could be accurately and rapidly detected and differentiated into different tissue types using the quadruplex sequencing assay.

## Introduction

The genus of *Bartonella* includes a group of Gram-negative bacteria that can infect a wide variety of mammals. A high prevalence of *Bartonella* spp. bacteremia has been reported in rodents, ruminants, cats, and many other animals throughout the world (Heller et al., 1997; Breitschwerdt and Kordick, 2000; Chang et al., 2000; Dehio et al., 2001; Ying et al., 2002; Chomel et al., 2006; Bai et al., 2011). *Bartonella* bacteria exhibit extremely high genetic diversity with more than 40 *Bartonella* species and subspecies originating from different mammalian species having been described. Several species have been recognized as emerging pathogens and have been associated with illness in human (Welch et al., 1992; Daly et al., 1993; Anderson and Neuman, 1997; Kerkhoff et al., 1999; Breitschwerdt and Kordick, 2000; Roux et al., 2000; Boulouis et al., 2005; Eremeeva et al., 2007; Kosoy et al., 2008). Many *Bartonella* species are host-specific, suggesting the maintenance of species in independent enzootic cycles (Vayssier-Taussat et al., 2009). On the other hand, coinfections with multiple *Bartonella* species in the same host species or even in an individual host have been observed (Bai et al., 2011; Qurollo et al., 2014; Melo et al., 2023). Some *Bartonella* species originating from the same mammalian order are closely related and form a species complex (Kosoy et al., 2012). For example, *Bartonella* species that are related to ruminants, such as *Bartonella melophagi* and *Bartonella schoenbuchensis*, which originated from sheep and roe deer, respectively, were genetically very similar to each other and belong to the *Bartonella bovis* species complex based on their DNA sequences for *gltA* (Kosoy et al., 2012). *Bartonella* bacteria are transmitted by hematophagous arthropods, such as fleas, flies, and lice, each of which is responsible for the transmission of different *Bartonella* species (Higgins et al., 1996; Maurin and Raoult, 1996; Fournier et al., 2001; Battisti et al., 2015). Continued investigation of the biology and epidemiology of bacteria belonging to this genus is necessary to determine the broad geographical distribution, the wide spectrum of reservoir hosts, and the zoonotic potential of *Bartonella* species.

Reliable detection methods are needed to facilitate ecological and epidemiological studies of *Bartonella* species. For the detection of *Bartonella* species, conventional polymerase chain reaction (PCR) and real-time PCR have been widely applied (Ciervo and Ciceroni, 2004; Angelakis et al., 2009; Morick et al., 2009; Colborn et al., 2010; Diaz et al., 2012), followed by Sanger sequencing to allow species identification (Šlapeta and Šlapeta, 2016; Zouari et al., 2017). Because Sanger sequencing only sequences one product, a PCR for amplifying different products needs to be performed separately to produce singular PCR amplicons to enable downstream sequencing. Moreover, if multiple strains are coinfecting a sample, Sanger sequencing may not yield clean and interpretable sequences. The traditional approach can be tedious, can be time- and reagent-consuming, and requires large volumes of DNA that can be problematic when dealing with very limited samples.

The application of innovative next-generation sequencing (NGS) techniques has solved these issues to a great extent and empowers scientists to conduct research in a rapid, accurate, and cost-effective manner. Compared with traditional PCR assays, NGS offers high sensitivity, offers increased specificity, and consumes fewer nucleic acids (Hojgaard et al., 2020). With increasing availability, NGS has become a popular tool to be used in a wide range of areas, from clinical microbiology to public health (Deurenberg et al., 2017; Hilt and Ferrieri, 2022). Recently, researchers have applied NGS for studies of *Bartonella* species in rodents and fleas (Gutiérrez et al., 2014; Himsworth et al., 2020; Power et al., 2021). Target *gltA* was used for the detection of *Bartonella* species in these studies. Power et al. (2021) included *ssrA* in addition to *gltA* in their study, with each of the two targets being amplified in a separate PCR reaction.

Owing to the extremely high genetic diversity within the *Bartonella* genus, using one gene target to detect all *Bartonella* species is challenging. Thus, a failure in detecting all *Bartonella* species may be due to solely relying on one genetic target. On the other hand, because of the close genetic similarity between some *Bartonella* species, especially those falling in the same species complex (Kosoy et al., 2012), using one genetic target to differentiate such species may result in misidentification. Therefore, the sequence of a second target should be provided for confirmation. Furthermore, molecular detection methods should be applicable for a diverse array of specimen types, such as arthropods and mammalian blood and organs. Due to inhibitory effects, a target may work well for a certain tissue type but become less efficient for other specimen types. Inclusions of multiple targets in an assay will increase the success of detecting the presence of *Bartonella* species and provide robust confirmation of the identity of specific *Bartonella* species by having multiple target sequences.

In the present study, we developed a quadruplex PCR amplicon next-generation sequencing assay using the Illumina MiSeq platform to detect and differentiate between each *Bartonella* species. Four gene targets, namely, *gltA, groEL, rpoB*, and *ssrA*, were amplified simultaneously in a single PCR reaction and sequenced on the same run on the MiSeq. These targets are considered reliable tools for distinguishing *Bartonella* species and have been frequently used in conventional PCR or real-time PCR assays (Renesto et al., 2001; Zeaiter et al., 2002; La Scola et al., 2003; Diaz et al., 2012; Bai et al., 2017; Poofery et al., 2022). Our objective was to obtain *Bartonella*-specific sequences for at least two targets as confirmation for the differentiation of a *Bartonella* species using the assay. In the study, we (1) compared the capabilities of the four targets to detect and differentiate *Bartonella* species when used individually or collectively by singular PCR amplicon sequencing or quadruplex PCR amplicon sequencing, (2) assessed the sensitivity and specificity of the quadruplex PCR amplicon sequencing, (3) examined the quadruplex PCR amplicon sequencing assay for its ability to discriminate co-occurring *Bartonella* species by testing DNA mixed from different *Bartonella* species to simulate coinfections; and (4) evaluated the performance of the quadruplex PCR amplicon sequencing assay on different naturally infected specimens by testing DNA derived from the field-collected rats and lice.

## Materials and methods

### Gene selection and primers

To identify primers for the assay, we screened 19 pairs of primers targeting *16S* rRNA, *ftsZ, gltA, groEL, hbpA, nuoG, ribC, rpoB, ssrA*, and ITS. Primers were designed using PrimerQuest™ Tool within Integrated DNA Technologies (https://www.idtdna.com/SciTools) or primer-BLAST program within NCBI [Primer designing tool (nih.gov)] or adapted from previously published assays (Relman et al., 1990; Norman et al., 1995; Birtles and Raoult, 1996; Zeaiter et al., 2002; Maggi and Breitschwerdt, 2005; Morick et al., 2009; Colborn et al., 2010; Diaz et al., 2012; Johnson et al., 2013; Oksi et al., 2013). Each primer pair was tested individually using the NGS sequencing. Sequences were obtained for all of the tested *Bartonella* species with four pairs of the primers, including 16S rRNAs (P12E and P12B), *gltA* (BhCS781.p and BhCS1137.n), *groEL* (groEL_1F and groEL_1R), and *ssrA* (primers ssrA_F and ssrA_R), and 23 sequences were obtained from the 24 tested *Bartonella* species with *rpoB* (prAPT0244 and prAPT0245). Sequence analysis showed that these sequences of each target were unique for each *Bartonella* species except for the 16S rRNA sequences, which were found to be identical for some species, suggesting that the 16S rRNA primers were not specific. Other primer pairs amplified/identified fewer *Bartonella* species (Supplementary Table S1). Based on the evaluation, the abovementioned four pairs of primers that amplify *Bartonella*-specific regions on *gltA, groEL, rpoB*, and *ssrA*, respectively, were selected for developing the quadruplex PCR amplicon sequencing assay (Table 1).

**Table 1:** Genes and the primer sequences (with the Illumina overhand adapter sequences) used for the quadplex PCR amplicon sequencing assay.

**Gene**	**Primer name**	**Primer sequences**	**Amplicon size**	**References**
*gltA*	BhCS781.p (ap,s)	TCGTCGGCAGCGTCAGATGTGTATAAGAGACAGGGGGACCAGCTCATGGTGG	338 bp	Norman et al., 1995
	BhCS1137.n (ap,s)	GTCTCGTGGGCTCGGAGATGTGTATAAGAGACAGAATGCAAAAAGAACAGTAAACA		
*groEL*	groEL_Forward	TCGTCGGCAGCGTCAGATGTGTATAAGAGACAGATAKCCACGATCAAACTGCAT	339 bp	This study
	groEL_Reverse	GTCTCGTGGGCTCGGAGATGTGTATAAGAGACAGTGTTGCGTGAAGTTGCTTCT		
*rpoB*	prAPT0244	TCGTCGGCAGCGTCAGATGTGTATAAGAGACAGGATGTGCATCCTACGCATTATGG	408 bp	Oksi et al., 2013
	prAPT0245	GTCTCGTGGGCTCGGAGATGTGTATAAGAGACAGAATGGTGCCTCAGCACGTATAAG		
*ssrA*	ssrA_Forward	TCGTCGGCAGCGTCAGATGTGTATAAGAGACAGGCTATGGTAATAAATGGACAATG	262 bp	This study
	ssrA_Reverse	GTCTCGTGGGCTCGGAGATGTGTATAAGAGACAGGCTTCTGTTGCCAGGTG		Diaz et al., 2012

### Definition for the detection and differentiation of *Bartonella* species

Two criteria were used for the definition: the presumptive presence of *Bartonella* species is confirmed if sequences of only one of the four targets were obtained; differentiation of *Bartonella* species is confirmed if sequences of at least two of the four targets were obtained.

### DNA template of *Bartonella* species and non-*Bartonella* species

A total of 24 *Bartonella* species (Table 2) were included for assay development. All *Bartonella* cultures were obtained from collections at the CDC's Bacterial Diseases Branch in Fort Collins, CO. Genomic DNA was prepared by heating a heavy suspension of microorganisms for 15 min at 95°C followed by centrifugation of the lysed cells for 1 min at 8,000 rpm. The supernatant was transferred to a clean centrifuge tube to be used as the DNA template. The DNA concentration of each template was measured using Invitrogen™ Qubit™ 4 Fluorometer dsDNA HS Assay (Fisher Scientific, Pittsburgh, PA), and 10-fold dilutions were prepared from 20 pg/μl to 0.002 fg/μl for use in different experiments. The *Bartonella* species included in this study are the following: *Bartonella alsatica, B. bovis, Bartonella clarridgeiae, Bartonella doshiae, Bartonella elizabethae, Bartonella grahamii, Bartonella henselae, Bartonella japonica, Bartonella koehlerae, B. melophagi, Bartonella phoceensis, Bartonella quintana, Bartonella rattimassiliensis, Bartonella rochalimae, B. schoenbuchensis, Bartonella silvicola, Bartonella tamiae, Bartonella taylorii, Bartonella tribocorum, Bartonella vinsonii* subsp. *arupensis, B. vinsonii* subsp. *berkhoffii, B. vinsonii* subsp. *vinsonii, Bartonella volans*, and *Bartonella washoensis*.

**Table 2:** *Bartonella* species used for assay development with Illumina sequencing results of each target with singular PCR amplicon sequencing and quadruplex PCR amplicon sequencing.

***Bartonella species***	**Animal reservoir**	**Vector**	**Singular PCR amplicon sequencing**				**Quadruplex PCR amplicons sequencing**				**Results**
			***gltA***	***ssrA***	***groEL***	***rpoB***	***gltA***	***ssrA***	***groEL***	***rpoB***	
*Bartonella alsatica*	Rabbits	Fleas? Ticks?	Yes	Yes	Yes	Yes	Yes	Yes	Yes	Yes	Nine species identified by four targets
*Bartonella bovis*	Cattle	Biting flies? Ticks?	Yes	Yes	Yes	Yes	Yes	Yes	Yes	Yes	
*Bartonella doshiae*	Field voles	Fleas? Ticks?	Yes	Yes	Yes	Yes	Yes	Yes	Yes	Yes	
*Bartonella grahamii*	Bank voles	Fleas? Ticks?	Yes	Yes	Yes	Yes	Yes	Yes	Yes	Yes	
*Bartonella henselae*	Cats	Fleas	Yes	Yes	Yes	Yes	Yes	Yes	Yes	Yes	
*Bartonella koehlerae*	Cats	Fleas	Yes	Yes	Yes	Yes	Yes	Yes	Yes	Yes	
*Bartonella melophagi*	Sheep	Sheep keds	Yes	Yes	Yes	Yes	Yes	Yes	Yes	Yes	
*Bartonella schoenbuchensis*	Roe deer	Biting flies? tIcks?	Yes	Yes	Yes	Yes	Yes	Yes	Yes	Yes	
*Bartonella silvicola*	Rats	Fleas? Ticks?	Yes	Yes	Yes	Yes	Yes	Yes	Yes	Yes	
*Bartonella phoceensis*	Rats	Fleas? Ticks?	Yes	Yes	Yes	Yes	Yes	x	Yes	Yes	Thirteen species identified by three targets
*Bartonella tribocorum*	Rats	Fleas? Ticks?	Yes	Yes	Yes	Yes	Yes	x	Yes	Yes	
*Bartonella quintana*	Human	Human body lice	Yes	Yes	Yes	Yes	Yes	Yes	x	Yes	
*Bartonella rochalimae*	Canids?	Fleas? Ticks?	Yes	Yes	Yes	Yes	Yes	Yes	x	Yes	
*Bartonella vinsonii* subsp. *arupensis*	Deer mice	Fleas? Ticks?	Yes	Yes	Yes	Yes	Yes	Yes	x	Yes	
*Bartonella vinsonii* subsp. *berkhoffii*	Coyotes	Fleas? Ticks?	Yes	Yes	Yes	Yes	Yes	Yes	x	Yes	
*Bartonella vinsonii* subsp. *vinsonii*	Meadow voles	Ear mites	Yes	Yes	Yes	Yes	Yes	Yes	x	Yes	
*Bartonella elizabethae*	Rats	Fleas?	Yes	Yes	Yes	Yes	Yes	Yes	Yes	x	
*Bartonella japonica*	Field mice	Unknown	Yes	Yes	Yes	Yes	Yes	Yes	Yes	x	
*Bartonella rattimassiliensis*	Rats	Unknown	Yes	Yes	Yes	Yes	Yes	Yes	Yes	x	
*Bartonella taylorii*	Wood mice	Fleas? Ticks?	Yes	Yes	Yes	Yes	Yes	Yes	Yes	x	
*Bartonella volans*	Fly squirrels	Fleas? Ticks?	Yes	Yes	Yes	Yes	Yes	Yes	Yes	x	
*Bartonella washoensis*	Ground squirrels	Fleas? Ticks?	Yes	Yes	Yes	Yes	Yes	Yes	Yes	x	
*Bartonella clarridgeiae*	Cats	Fleas	Yes	Yes	Yes	x	Yes	Yes	x	x	Two species identified by two targets
*Bartonella tamiae*	Unknown	Unknown	Yes	Yes	Yes	Yes	Yes	Yes	x	x	

Three replicates of each sample were tested. Yes, identical sequences of the target for the *Bartonella* species were obtained for all three replicates; x, sequences of the target for the *Bartonella* species were obtained in ≤ 2 replicates.

DNAs from 19 different bacterial species that might be encountered in the field-collected vector or host specimens were used for specificity testing of the assay: *Anaplasma phagocytophilum, Bacillus anthracis, Borrelia burgdorferi, Borrelia mayonii, Borrelia miyamotoi, Brucella canis, Burkholderia cenocepacia, Escherichia coli, Francisella tularensis, Leptospira interrogans, Listeria monocytogenes, Rickettsia sibirica, Salmonella enterica, Staphylococcus aureus, Toxoplasma gondii, Trypanosoma cruzi, Yersinia pestis, Yersinia enterocolitica*, and *Yersinia pseudotuberculosis*. DNA templates of the abovementioned species were obtained from BEI Resources or the CDC Bacterial Diseases Branch.

### Singular PCR amplicon sequencing and quadruplex PCR amplicon sequencing

To evaluate the performance of the assay, the four pairs of primers were tested individually and collectively by singular PCR amplicon sequencing and quadruplex PCR amplicon sequencing. The procedures mainly include four steps: (1) primary PCR amplification with one or four pairs of the primers and purification; (2) indexing PCR and purification; (3) pooling, purification, quantification, and normalization; and (4) library denaturing and sample loading on Illumina Miseq (Illumina, San Diego, CA) to start sequencing. The detailed procedures can be referred from the previously published study by Hojgaard et al. (2020) and manufacturer's protocol with some modifications. A brief description is provided below.

Primary PCR: PCR reaction mixture were set up using one pair of primers or four pairs of primers. The reaction mixture contained 12.5 μl of Multiplex TEMPase 2x Master Mix (Amerigo Scientific, Central Islip, NY), forward and reverse primers (one pair or four pairs) each at a final concentration of 300 nM, 10 pg of DNA template, and nuclease-free water until the volume of 25 μl is achieved. Three replicates of each DNA template were tested in both singular PCR and quadruplex PCR. Targeted sequences must be obtained from all three replicates to finally call a sample positive. The PCR was carried out on a C1000 Touch thermal cycler (Bio-Rad, Hercules, CA) with the following conditions: denaturation at 95°C for 15 min, followed by 35 cycles of 95°C for 30 s, 58°C for 30 s, and 64°C for 60 s, and ending with incubation for 5 min at 72°C. Positive and negative controls were included in each PCR run to evaluate performance and detect contamination. Following amplification, the PCR products were purified with AMpure XP magnetic beads (Beckman Coulter, Brea, CA) and washed with 80% ethanol on a KingFisher Flex purification system (Thermo Fisher Scientific, Waltham, MA).

Index PCR: The reaction mixture contained 25 μl of Multiplex TEMPase 2x Master Mix (Amerigo Scientific, Central Islip, NY), 5 μl each of dual unique barcoded indices (Nextera XT Index Kit V2, Illumina, San Diego, CA), 5 μl purified PCR products from the primary PCR, and nuclease-free water until the volume of 50 μl was achieved. The PCR was carried out on a C1000 Touch Thermal Cycler (Bio-Rad, Hercules, CA) with the following conditions: denaturation at 98°C for 15 min followed by 12 cycles of 98°C for 20 s, 55°C for 20 s, and 68°C for 60 s, ending with an incubation of 5 min at 68°C. Following the indexing, the PCR products were purified with MagSi-DNA allround magnetic beads (BOCA Scientific, Westwood, MA), sodium acetate (3 M), and isopropanol and washed with 80% ethanol on a KingFisher Flex purification system (Thermo Fisher Scientific, Waltham, MA).

Pooling, purification, and quantification and normalization: the samples were indexed after the index PCR. All indexed samples were pooled, then purified with AMpure XP magnetic beads (Beckman Coulter, Brea, CA), and washed with 80% ethanol. Then, the concentration was measured using Invitrogen™ Qubit™ 4 Fluorometer dsDNA HS Assay (Fisher Scientific, Pittsburgh, PA). The final concentration was quantified and normalized based on the amplicon size.

Denaturing and sample loading on Miseq Illumina to start sequencing: the library from the previous step was denatured with 0.1 N NaOH and then heat denatured. PHIX was added as an internal control. The library was loaded into an MiSeq Nano v2 (500 cycles) reagent cassette (Illumina, San Diego, CA) to start sequencing on an Illumina Miseq instrument (Illumina, San Diego, CA).

### Sensitivity and specificity assessment

Sensitivity and specificity were tested to assess the performance of the quadruplex PCR amplicon sequencing assay. DNA of *B. grahamii* and *B. koehlerae* with concentrations of 100 pg−0.01 fg was tested to estimate the sensitivity. The reasons we picked *B. grahamii* and *B. koehlerae* were because the two species were among those that worked well with both singular and quadruplex sequencing, and animal reservoirs of the two species (rodents and cats, respectively) are commonly studied by researchers. Three replicates of each concentration were tested. All samples with different concentrations were pooled, purified, final concentration normalized, and sequenced. The lowest dilution that was able to generate targeted sequences in all three replicates in the quadruplex sequencing was considered the limit of detection.

The specificity of the assay was assessed by testing high concentrations (500 pg) of DNA from 19 non-*Bartonella* bacterial species listed in the subsection “DNA template of *Bartonella* species and non-*Bartonella* species.” Three replicates of each DNA template were tested. *Bartonella* sequences must be absent in any of the three replicates of any sample to be called specific.

### Application of the assay for the detection and differentiation of co-occurring *Bartonella* species

Because different *Bartonella* species may co-occur in the same host species or in the same individual (Bai et al., 2011; Qurollo et al., 2014; Melo et al., 2023), we evaluated the quadruplex sequencing assay for its ability to accurately identify co-occurring *Bartonella* species by testing DNA mixed from different *Bartonella* species to simulate coinfections. The mixed DNA included (1) *B. henselae* and *B. koehlerae*, both of which are cat-associated and can coinfect the same individual (Qurollo et al., 2014; Melo et al., 2023), and (2) *B. henselae, B. koehlerae*, and *B. grahamii* (rodent-associated), which was added to increase the degree of difficulty for species resolution. Three replicates of each mixed DNA template were tested with 10 pg DNA of each *Bartonella* species. Targeted sequences must be obtained from all three replicates to finally call a sample positive.

### Application of the assay for testing of field samples

To evaluate the utility of the quadruplex sequencing assay in naturally infected specimens, we tested DNA extracted from field-collected samples. Specimens included human body lice (*Pediculus humanus humanus, n* = 40 pools, 10 lice/pool). All lice first were tested by conventional PCR targeting *gltA* followed by Sanger sequencing for species identification using a previously described assay (Norman et al., 1995). The quadruplex sequencing was then performed and compared to the conventional PCR results. We also tested spleens from Norway rats (*Rattus norvegicus*, n=54). The rat spleens were partial samples from a previous study, which reported *B. tribocorum* in these rats with a prevalence range of 0–60% through blood culturing (Himsworth et al., 2015). All lice DNA and rat spleen DNAs were obtained from collections at the CDC's Bacterial Diseases Branch.

### Bioinformatics analysis

After sequencing was completed, the raw sequences were analyzed with a custom Nextflow bioinformatics pipeline described by Osikowicz et al. (2023) and is publicly available on GitHub [CDCgov/tick_surveillance (github.com)] with detailed instructions. Briefly, the raw sequences, primer information, reference sequences, and sample data were input to the pipeline. The pipeline first performed quality control analysis with FastQC version 0.11 (Andrews, 2010) and primer trimming with Cutadapt version 3.5 (Martin, 2011). Only reads with the appropriate primer combinations were trimmed and proceeded to the next step. Then, DADA2 version 1.18 (Callahan et al., 2016) performed error correction, removed chimeric sequences, merged paired reads, grouped amplicon sequence variants (ASV), and calculated ASV abundance. The observed ASVs were then aligned to reference sequences with BLASTn version 2.10.0 (Altschul et al., 1990; Camacho et al., 2009). The minimum read cutoffs for a sample to be considered positive was set to be 50 reads. A 90% sequence similarity and 90% minimum sequence alignment length was used to align the observed ASVs to the reference sequences. Sequences that represent different *Bartonella* species for each target were obtained from the GenBank database and used as reference sequences. If a *Bartonella* species sequence for a target was not available in GenBank, then the sequence obtained from this study was used as a reference sequence. Finally, a summary report was generated and any unassigned ASVs were searched against the NCBI nucleotide database with BLASTn, and then unique sequences were submitted to GenBank.

All sequences generated from the current work for each target were further compared between themselves using the Clustal V program within the MegAlign module of DNASTAR Lasergene 17 (DNASTAR, Madison, WI). A phylogenetic tree was constructed for each gene using the neighbor-joining method to compare the genetic similarity of the *Bartonella* species.

## Results

### Singular PCR amplicon sequencing

In the singular PCR amplicon sequencing setting in which DNA templates were amplified with one pair of the primers in the primary PCR reaction, amplicon sequences of *gltA, groEL*, and *ssrA* were obtained for all 24 *Bartonella* species in all three replicates and amplicon sequences of *rpoB* were obtained for 23 of the 24 *Bartonella* species except for *B. clarridgeiae* in all three replicates (Table 2). The pipeline alignment analysis with the reference sequences demonstrated each amplicon sequence for each of all four targets obtained in the present study, which was of the expected *Bartonella* species. For *B. volans*, the *rpoB* sequence was identical to the reference (EU294520). There was no reference available for *gltA, groEL*, and *ssrA*, and thus, the sequences for *gltA, groEL*, and *ssrA* obtained in the current work were novel and were deposited in GenBank with accession numbers OR072638, OR072639, and OR072640, respectively.

**Figure 1:**
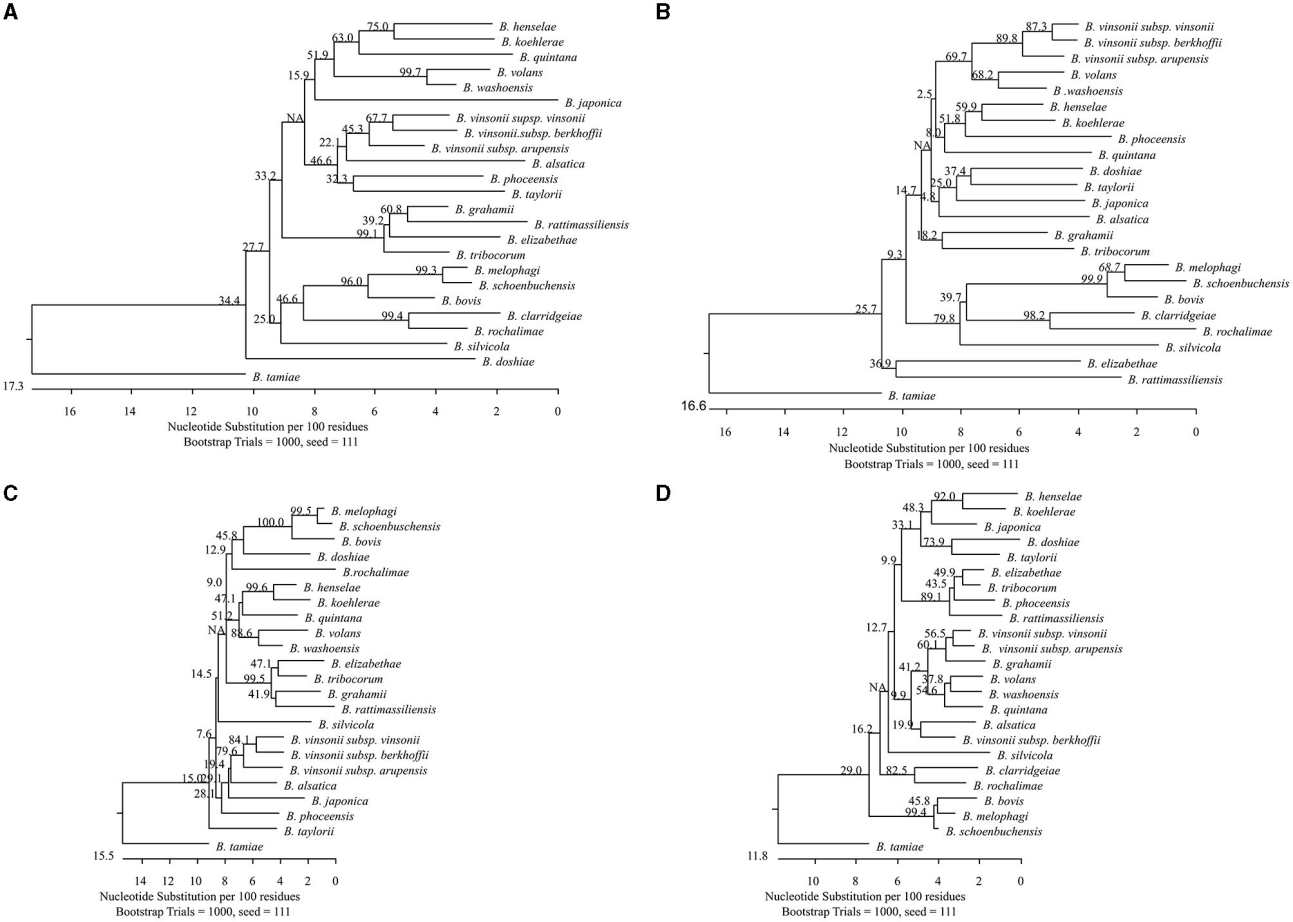
Phylogenetic relationships of the 24 *Bartonella* species tested in this study based on partial sequences of *gltA* **(A)**, *groEL* **(B)**, *rpoB* **(C)**, and *ssrA* **(D)**. The *Bartonella* species were separated by each target with genetic divergence of 1.8%−25.6%, 3.8%−24.3%, 1.8%−22.4%, and 1.2%−16.6%, for *gltA, groEL, rpoB*, and *ssrA*, respectively. *Bartonella melophagi* and *Bartonella schoenbuchensis*, belonging to the *Bartonella bovis* species complex, had smaller divergence between themselves. The *groEL* sequences better separated the two species with higher divergence (3.8%). The phylogenetic tree was constructed by the neighboring-joining method, and bootstrap values were calculated with 1,000 replicates.

Phylogenetic analysis of the sequences showed that all tested *Bartonella* species were separated by each target with genetic divergence of 1.8%−25.6%, 3.8%−24.3%, 1.8%−22.4%, and 1.2%−16.6%, for *gltA, groEL, rpoB*, and *ssrA*, respectively (Figures 1A–D). *Bartonella melophagi* and *B. schoenbuchensis* were close with smaller divergence by *gltA, rpoB*, and *ssrA*; *groEL* sequences showed more divergence (3.8%).

### Quadruplex PCR amplicon sequencing

In the quadruplex PCR amplicon sequencing setting in which DNA templates were amplified with all four pairs of primers simultaneously in the primary PCR reaction, of the 24 tested *Bartonella* species, amplicon sequences of *gltA, ssrA, groEL*, and *rpoB* were obtained for 24, 22, 17, and 16 species, respectively, in three of the three replicates (Table 2). The median numbers of normalized reads per sample were 615 (range 51–1,374), 270 (range 54–864), 303 (range 53–748), and 103 (range 50–378) for *gltA, ssrA, groEL*, and *rpoB*, respectively. The *ssrA* sequences were not obtained for two *Bartonella* species (*B. phoceensis* and *B. tribocorum*); the *groEL* sequences were not obtained for seven *Bartonella* species (*B. clarridgeiae, B. quintana, B. rochalimae, B. tamiae, B. vinsonii* subsp. *arupensis, B. vinsonii* subsp. *berkhoffii*, and *B. vinsonii* subsp. *vinsonii*); and the *rpoB* sequences were not obtained for eight *Bartonella* species (*B. clarridgeiae, B. elizabethae, B. japonica, B. rattimassiliensis, B. tamiae, B. taylorii, B. volans*, and *B. washoensis*; Table 2). These results suggested that *gltA* and *ssrA* work better than *groEL*, followed by *rpoB* in the quadruplex PCR amplicon sequencing.

Some amplicon sequences were obtained from one or two of the three triplicates for some *Bartonella* species/gene targets within the quadruplex amplicon sequencing. Because we only counted sequences which were obtained in all three replicates, those with one or two of the three replicates were still considered negative.

Overall, using the quadruplex amplicon sequencing with targets *gltA, groEL, rpoB*, and *ssrA*, all 24 *Bartonella* species each were correctly classified with confirmation by at least two target sequences (our criterion by definition). Among those, nine *Bartonella* species were identified with all four targets; 13 *Bartonella* species were identified with three targets; and two *Bartonella* species were identified with two targets (Table 2).

### Sensitivity and specificity testing

Testing DNA of *B. grahamii* and *B. koehlerae* with concentrations of 100 pg−0.01 fg using the quadruplex amplicon sequencing assay, specific sequences for all four targets were obtained in all three replicates with DNA concentrations ≥1 fg/reaction for each target for both *B. grahamii* and *B. koehlerae*. The limit of detection was determined as 1 fg/reaction for the two species, which also was considered the limit of detection of the assay.

For specificity testing, no *Bartonella* sequences were observed from any of the 19 non-*Bartonella* bacterial species (three replicates each) using the quadruplex amplicon sequencing. Gel electrophoresis also showed no presence of *Bartonella* amplicons for each target (Supplementary Figures S1–S4).

### Application of the assay for the detection and differentiation of co-occurring *Bartonella* species

Using the quadruplex sequencing assay to screen the DNA mix of *B. henselae* and *B. koehlerae*, both *B. henselae* and *B. koehlerae* were successfully discriminated by all four targets with species-specific sequences obtained in all three replicates. From the DNA mix of *B. henselae, B. koehlerae*, and *B. grahamii*, the three *Bartonella* species were discriminated by *rpoB* and *ssrA* with species-specific sequences obtained in all three replicates. *gltA* sequences specific to *B. henselae* and *B. grahamii* were obtained in all three replicates but not for *B. koehlerae. groEL* sequences specific to *B. henselae* and *B. koehlerae* were obtained in all three replicates but not for *B. grahamii* (Table 3). Although not all targets worked, all of the three *Bartonella* species known in the DNA mix were accurately identified with sequence confirmation by at least three targets, which met or surpassed our criterion of species differentiation.

**Table 3:** Discrimination of co-occurred *Bartonella* species using the quadruplex PCR amplicon sequencing.

	***Bartonella henselae** + **Bartonella koehlerae***		***Bartonella henselae** + **Bartonella koehlerae** + **Bartonella grahamii***		
	***Bartonella henselae***	***Bartonella koehlerae***	***Bartonella henselae***	***Bartonella koehlerae***	***Bartonella grahamii***
*gltA*	Yes	Yes	Yes	x	Yes
*groEL*	Yes	Yes	Yes	Yes	x
*rpoB*	Yes	Yes	Yes	Yes	Yes
*ssrA*	Yes	Yes	Yes	Yes	Yes

Three replicates of each sample were tested. Yes, identical sequences of the target for the *Bartonella* species were obtained for all three replicates. x, sequences of the target for the *Bartonella* species were obtained in ≤ 2 replicates.

### Application of the assay for testing of field samples

Testing of DNA obtained from lice: conventional PCR targeting *gltA* showed that all lice (*n* = 40 pools, 10 lice/pool) were positive for *Bartonella* and was identified as *B. quintana* by sequencing. Using the quadruplex amplicon sequencing assay, we obtained sequences of *gltA, rpoB*, and *ssrA* from all 40 pools and all sequences were identified correctly as *B. quintana*. No *groEL* sequences were obtained. This was expected as the *groEL* primers did not amplify *B. quintana* DNA derived from culture (Table 4). The results using the quadruplex sequencing assay are in accordance with that.

**Table 4:** Performance comparison of the quadruplex PCR amplicon sequencing on *Bartonella quintana-* and *B. triborcorum-*DNA derived from different sample types.

	**Culture-derived *Bartonella* quintana DNA**	**Lice-derived *Bartonella* quintana DNA**	**Culture-derived *Bartonella* tribocorum DNA**	**Rat spleen-derived *Bartonella tribocorum DNA***
*gltA*	Yes	Yes	Yes	Yes
*groEL*	x	x	Yes	Yes
*rpoB*	Yes	Yes	Yes	x
*ssrA*	Yes	Yes	x	x

Three replicates of each sample were tested. Yes, identical sequences of the target for the *Bartonella* species were obtained for all three replicates. x, sequences of the target for the *Bartonella* species were obtained in ≤ 2 replicates.

Testing of DNA obtained from rat spleens: using the quadruplex amplicon sequencing assay, 54 DNA extracts derived from rat spleens were tested for *Bartonella* species. Sequences for *gltA* and *groEL* were obtained from 33 (61.1%) samples. All sequences of *gltA* and *groEL* were identified as *B. tribocorum*. The results were consistent with findings reported in an earlier study on these rats through blood culturing (Himsworth et al., 2015). No *ssrA* sequences were obtained. This was expected because the *ssrA* primers did not amplify *B. tribocorum* DNA derived from culture. However, no sequences of *rpoB* were obtained from the rat spleen DNA as well, suggesting a difference between rat spleen-derived DNA and culture-derived DNA (Table 4).

## Discussion

The significant genetic diversity within the *Bartonella* genus presents a challenge for molecular detection of all *Bartonella* species using a single target. PCR may fail because of primer binding site polymorphisms. In addition, the genetic similarity between some *Bartonella* species such as those within a species complex makes them indistinguishable using a single target because of the limited genetic divergence. Using multiple targets can increase the success of detection and differentiation of *Bartonella* species. Here we developed a quadruplex PCR amplicon next generation sequencing assay by incorporating *gltA, groEL, rpoB*, and *ssrA* using the Illumina MiSeq platform for detection and differentiation of *Bartonella* species. None of the included reference species that might be found in field-collected specimens were falsely classified as *Bartonella* in our assay and each of the detected *Bartonella* species were correctly assigned to species. The assay had a low limit of detection identified as 1 fg/reaction based on the testing of *B. grahamii* and *B. koehlerae*. This was very comparable to those reported in real-time PCR (Diaz et al., 2012). Nevertheless, the sensitivity may vary for different *Bartonella* species.

The four targets used in the assay were evaluated for their capability to differentiate *Bartonella* species used in singular amplicon sequencing or quadruplex amplicon sequencing. It was observed that the efficiency of the targets differed when used individually (singular amplicon sequencing) or collectively (quadruplex amplicon sequencing). When used individually, all *Bartonella* species were detected by the four targets, except for *B. clarridgeiae*, which was not detected by *rpoB*, which could be related to the sequence variants within primer binding site. These targets became less efficient in the quadruplex amplicon sequencing. For example, *B. quintana groEL* sequences were detected when used in singular amplicon sequencing but not detected in quadruplex amplicon sequencing. Similarly, *B. tribocorum ssrA* sequences were detected in singular amplicon sequencing but not in quadruplex amplicon sequencing. Overall, *gltA* and *ssrA* were more efficient than *groEL* and *rpoB* in the quadruplex amplicon sequencing. When amplifying multiple amplicons simultaneously in a single PCR, the amplification efficiency of each of the targets may be affected by different factors such as primer interference, product size, primer concentration, and cycling conditions. We optimized cycling conditions in this study (data not shown) but used the standard parameters for others. Additional optimization on different factors (such as primer concentration) may improve the performance of the assay. Nevertheless, all 24 *Bartonella* species tested in the study were successfully differentiated using the quadruplex amplicon sequencing assay with confirmation of sequences for at least two targets that met our criterion of species differentiation. In fact, among the 24 tested *Bartonella* species, except for two species, that were differentiated with only two targets, all others were differentiated with four (9/24) or three targets. Some amplicon sequences were obtained from one or two of the three triplicates for some *Bartonella* species/gene targets with the quadruplex amplicon sequencing, but these sequences were still considered negative since we only counted those with sequences obtained in all three replicates. When only one or two of the three replicates yield a positive result, retesting with singular amplicon sequencing assays might build confidence in calling a sample positive.

Using the quadruplex amplicon sequencing assay, *Bartonella* species that are extremely genetically similar were better differentiated with additional data. Among the *Bartonella* species we tested, *B. melophagi* and *B. schoenbuchensis*, originating from sheep and roe deer, respectively, belong to the ruminant-associated *B. bovis* species complex (Kosoy et al., 2012). The two *Bartonella* species were very closely related by *gltA, rpoB*, and *ssrA* (1.2%−1.8% divergence). It would be very difficult to define them as different species according to La Scola et al. (2003), who proposed using 96% sequence similarity to define species. The *groEL* sequences, however, showed 3.8% divergence between the two species. This helped to differentiate the two species. Most importantly, the original description of the two species was linked mainly to their host association (Dehio et al., 2001; Kosoy et al., 2016) rather than the genetic differences.

Co-occurrence of multiple *Bartonella* species in their hosts has been reported (Bai et al., 2011; Qurollo et al., 2014; Melo et al., 2023). We mixed DNA from different *Bartonella* species mimicking the natural occurrence of coinfections. Testing the DNA mix showed that co-occurring *Bartonella* species were accurately identified using the quadruplex amplicon sequencing assay, although the efficiency decreased when more numbers of *Bartonella* species are present.

We tested DNA extracted from human body lice and spleens of Norway rats using the quadruplex amplicon sequencing assay to evaluate its performance in naturally infected specimens. *Bartonella quintana* was detected in all lice with sequence confirmation by three targets except for *groEL*. The other three targets showed consistent efficiency in the testing of lice- and culture-derived *B. quintana* DNAs. From the rats, *B. tribocorum* was detected in 61.1% of the samples with sequence confirmation by *gltA* and *groEL*. The prevalence was comparable with an earlier report on these rats through blood culturing (Himsworth et al., 2015). Interestingly, in the quadruplex amplicon sequencing, *rpoB* primers did not detect *Bartonella* DNA in any of the rat spleen samples. This was surprising because *rpoB* worked well when testing culture-derived *B. tribocorum* DNA. When applying a molecular method for pathogen detection, inhibition is often a problem, which sometimes causes a false-negative detection (Schrader et al., 2012). The inhibiting substances may be present in the analyzed samples and may affect the sensitivity of an assay (Kaneko et al., 2007; Nkouawa et al., 2010). Previous studies have reported the presence of inhibiting substances from DNAs of mouse liver, spleen, and lung in PCR (Feng et al., 2018; Bai et al., 2022). Our results also may suggest the presence of inhibiting substances from spleen-derived DNAs that have occurred in the testing. However, the inhibition seemed only to affect *rpoB* but not *groEL* or *gltA*, highlighting the advantage of a multi-target assay. A strategy to remove the inhibiting effects may be adapted for better outcomes. Nevertheless, with sequence confirmation by *gltA* and *groEL*, we were able to detect and differentiate *B. tribocorum* in the rats using the quadruplex amplicon sequencing assay.

In conclusion, we have developed a highly sensitive and specific quadruplex PCR amplicon sequencing assay using the NGS technology for the detection and differentiation of *Bartonella* species. Because the four targets varied in efficiency for different *Bartonella* species, knowing what *Bartonella* species to expect when applying the assay would be helpful. Our study also provided valuable insights into the application of the assay for different sample type, which highlights the need to evaluate assay performance when testing alternative sample types.

## Data availability statement

The original contributions presented in the study are publicly available. This data can be found here: National Center for Biotechnology Information (NCBI) GenBank, https://www.ncbi.nlm.nih.gov/genbank/, OR072638, OR072639, and OR072640.

## Author contributions

YB conceived, designed, and performed the experiment, analyzed the data, and wrote the paper. LO analyzed the data and reviewed the paper. AH designed and reviewed the paper. RE supervised, reviewed, and edited the paper. All authors contributed to the article and approved the submitted version.

## Supplementary material

Supplementary Table S1 Genes and the primer sequences (with the Illumina overhand adapter sequences) evaluated and sequencing results.

Supplementary Figure S1 Gel electrophoresis of *gltA*, Number 1–24 were the 24 *Bartonella* species (listed in Table 2) and number 25–43 were the 19 non-*Bartonella* species (listed in Section “DNA template of *Bartonella* species and non-*Bartonella* species) tested in the study. All *Bartonella* species was amplified and none of the non-*Bartonella* species was amplified.

Supplementary Figure S2 Gel electrophoresis of *ssrA*. Number 1–24 were the 24 *Bartonella* species (listed in Table 2) and number 25–43 were the 19 non-*Bartonella* species (listed in Section “DNA template of *Bartonella* species and non-*Bartonella* species) tested in the study. All *Bartonella* species were amplified and none of the non-*Bartonella* species was amplified.

Supplementary Figure S3 Gel electrophoresis of *groEL*, Number 1–24 were the 24 *Bartonella* species (listed in Table 2) and number 25–43 were the 19 non-*Bartonella* species (listed in Section “DNA template of *Bartonella* species and non-*Bartonella* species) tested in the study. All *Bartonella* species was amplified and none of the non-*Bartonella* species was amplified.

Supplementary Figure S4 Gel electrophoresis of *rpoB*, Number 1–24 were the 24 *Bartonella* species (listed in Table 2) and number 25–43 were the 19 non-*Bartonella* species (listed in Section “DNA template of *Bartonella* species and non-*Bartonella* species) tested in the study. All *Bartonella* species but *B. clarridgeiae* (#23) was amplified and none of the non-*Bartonella* species was amplified.
