# Corrigendum: Development of a quadruplex PCR amplicon next generation sequencing assay for detection and differentiation of *Bartonella* spp.

**DOI:** 10.3389/fmicb.2024.1360286

**Published:** 2024-02-26

**Authors:** Ying Bai, Lynn M. Osikowicz, Andrias Hojgaard, Rebecca J. Eisen

**Affiliations:** Bacterial Disease Branch, Division of Vector-Borne Diseases, Centers for Disease Control and Prevention, Fort Collins, CO, United States

**Keywords:** assay development, quadruplex, next-generation sequencing (NGS), *Bartonella* spp., differentiation

In the published article, there was an error in the supplementary material. Supplementary Figure S3 was identical to Supplementary Figure S1, but should have been a different image. The file for Supplementary Figure S3 has been replaced with the correct image. The correct Supplementary Figure S3 appears below:

**Supplementary Figure S3:**
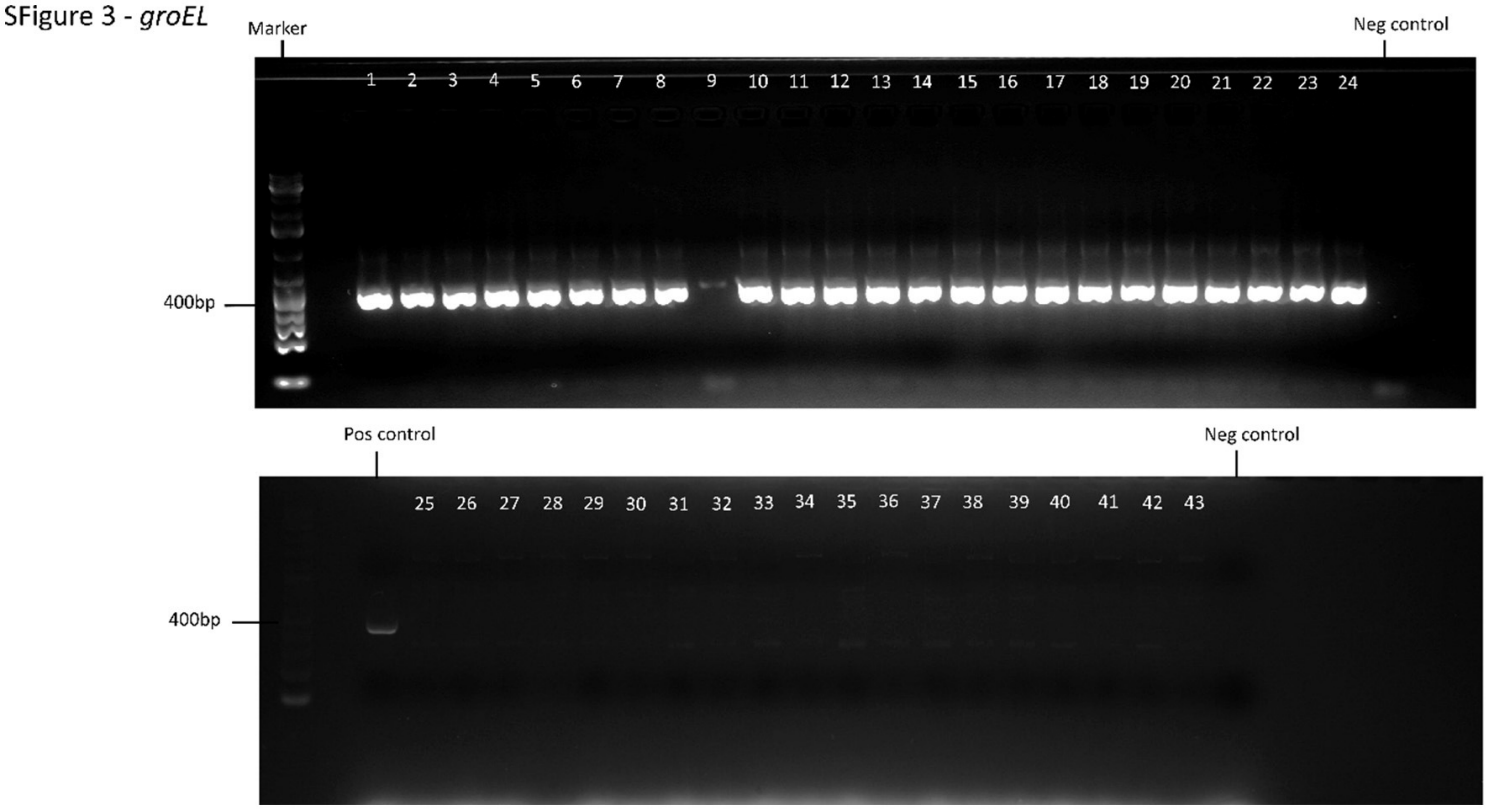


The authors apologize for this error and state that this does not change the scientific conclusions of the article in any way. The original article has been updated.

