# Supplementary material for: Development of a quadruplex PCR amplicon next generation sequencing assay for detection and differentiation of Bartonella spp
Source: Front Microbiol. 2023 Dec 7;14:1243471. doi: 10.3389/fmicb.2023.1243471 (PMC10733521; doi:10.3389/fmicb.2023.1243471)
Supplement: Supplementary Table S1 — Genes and the primer sequences (with the Illumina overhand adapter sequences) evaluated and sequencing results. [file Data_Sheet_1.PDF]

S1 Table. Genes and the primer sequences (with the Illumina overhand adapter sequences) evaluated and sequencing results.

Gene	Primer name	Primer sequences	Amplicon size	Testing results/notes	Reference/Source
16S rRNA	P24E	TCGTCGGCAGCGTCAGATGTGTATAAGAGACAGGGAATTCCCTCCTTCAGTTAGGCTGG	241bp	Seqeunces were obtained for all tested *Bartonella* species, but non-specific.	Relman et al., 1990
	P12B	GTCTCGTGGGCTCGGAGATGTGTATAAGAGACAGCGGGATCCCGAGATGGCTTTTGGAGATTA			
ftsZ	ftsZ_1F	TCGTCGGCAGCGTCAGATGTGTATAAGAGACAGAAACAGCGGAAGCTGGTATAG	403bp	Sequences were obtained for 4 of the 24 tested species.	This study
	ftsZ_1R	GTCTCGTGGGCTCGGAGATGTGTATAAGAGACAGGCCTCATCCACTTCAAACAAAG			
ftsZ	ftsZ_2F	TCGTCGGCAGCGTCAGATGTGTATAAGAGACAGAAACrGCrGArdCTGGyAywG	403bp	Sequences were obtained for 11 of the 24 tested species.	This study
	ftsZ_2R	GTCTCGTGGGCTCGGAGATGTGTATAAGAGACAGGCyTCATCnACyTCrAAnArrG			
ftsZ	ftsZ_3F	TCGTCGGCAGCGTCAGATGTGTATAAGAGACAGGCTGATGTTCGTTCTGTTATGC	210bp	Sequences were obtained for 3 of the 24 tested species.	This study
	ftsZ_3R	GTCTCGTGGGCTCGGAGATGTGTATAAGAGACAGATTAGCAGCCTCATCCACTTC			
ftsZ	ftsZ_4F	TCGTCGGCAGCGTCAGATGTGTATAAGAGACAGGCwGAyGTTCGyTCTGTTATGC	210bp	Sequences were obtained for 6 of the 24 tested species.	This study
	ftsZ_4R	GTCTCGTGGGCTCGGAGATGTGTATAAGAGACAGATTAGChGCyTCATCnACyTC			
gltA	BhCS781.p (ap,s)	TCGTCGGCAGCGTCAGATGTGTATAAGAGACAGGGGGACCAGCTCATGGTGG	338bp	Sequences were obtained for all tested *Bartonella* species.	Norman et al., 1995
	BhCS1137.n (ap,s)	GTCTCGTGGGCTCGGAGATGTGTATAAGAGACAGAATGCAAAAAGAACAGTAAACA			
gltA	443F	TCGTCGGCAGCGTCAGATGTGTATAAGAGACAGGCTATGTCTGCATTCTATCA	340bp	Sequences were obtained for 4 of the 24 tested species.	Birtle, and Raoult, 1996<br>Norman et al., 1995
	781R	GTCTCGTGGGCTCGGAGATGTGTATAAGAGACAGCCACCATGAGCTGGTCCCC			
groEL	groEL_1F	TCGTCGGCAGCGTCAGATGTGTATAAGAGACAGATAKCCACGATCAAACTGCAT	339bp	Sequences were obtained for all tested *Bartonella* species.	This study
	groEL_1R	GTCTCGTGGGCTCGGAGATGTGTATAAGAGACAGTGTTGCGTGAAGTTGCTTCT			
groEL	groEL_2F	TCGTCGGCAGCGTCAGATGTGTATAAGAGACAGGCTTCACCTTCCACATCTTC	157bp	Sequences were obtained for 22 of the 24 tested *Bartonella* species.	This study
	groEL_2R	GTCTCGTGGGCTCGGAGATGTGTATAAGAGACAGATGCAGTTTGATCGTGGATA			
groEL	HSP1 (s)	TCGTCGGCAGCGTCAGATGTGTATAAGAGACAGGGAAAAAGTNGGCAATGGAG	365bp	Sequences were obtained for 6 of the 24 tested *Bartonella* species.	Zeaiter et al., 2002
	HSPS1 (s)	GTCTCGTGGGCTCGGAGATGTGTATAAGAGACAGAAGCNCCNGGNTTTGGTGA			
hbpA	hbpA_F	TCGTCGGCAGCGTCAGATGTGTATAAGAGACAGCTATATTGGTGGTCAAGTTG	386bp	Sequences were obtained for 5 of the 24 tested *Bartonella* species.	This study
	hbpA_R	GTCTCGTGGGCTCGGAGATGTGTATAAGAGACAGCCAGCAACATAAGGCATAAT			
ITS	ITS_F	TCGTCGGCAGCGTCAGATGTGTATAAGAGACAGGCTGGATCACCTCCTTTCTAAG	266bp	Sequences were obtained for 13 of the 24 tested species.	This study
	ITS_R	GTCTCGTGGGCTCGGAGATGTGTATAAGAGACAGCCAGAAGGCTTGGGATCATCATCT			
ITS	321s	TCGTCGGCAGCGTCAGATGTGTATAAGAGACAGAGATGATGATCCCAAGCCTTCTGG	190bp	Sequences were obtained for 9 of the 24 tested species.	Maggi, and Breitschwerdt, 2005
	H493as	GTCTCGTGGGCTCGGAGATGTGTATAAGAGACAGTGAACCTCCGACCTCACGCTTATC			

nuoG	nuoG_F	TCGTCGGCAGCGTCAGATGTGTATAAGAGACAGGGCGTGATTGTTCTCGTTA	347bp	Sequences were obtained for 4 of the 24 tested species.	Colborn et al., 2010
	nuoG_R	GTCTCGTGGGCTCGGAGATGTGTATAAGAGACAGCACGACCACGGCTATCAAT			
ribC	BARTON-1_F	TCGTCGGCAGCGTCAGATGTGTATAAGAGACAGTAACCGATATTGGTTGTGTTGAAG	297bp	Sequences were obtained for 3 of the 24 tested species.	Johnson et al., 2013
	ribC_1R	GTCTCGTGGGCTCGGAGATGTGTATAAGAGACAGCACGTCGTCATTTCAAGTGTATG			This study
ribC	BARTON-1_F	TCGTCGGCAGCGTCAGATGTGTATAAGAGACAGTAACCGATATTGGTTGTGTTGAAG	297bp	Sequences were obtained for 1 of the 24 tested species.	Johnson et al., 2013
	ribC_2R	GTCTCGTGGGCTCGGAGATGTGTATAAGAGACAGCNCGTYGTCAYTTYRAGYGTRTG			This study
rpoB	600f	TCGTCGGCAGCGTCAGATGTGTATAAGAGACAGGAAAATGATGATGCGAATCG	210bp	Sequences were obtained for 20 of 24 tested *Bartonella* species.	Morick et al., 2009
	800r	GTCTCGTGGGCTCGGAGATGTGTATAAGAGACAGGATCTAAATCTTCTGTTGCACG			
rpoB	prAPT0244	TCGTCGGCAGCGTCAGATGTGTATAAGAGACAGGATGTGCATCCTACGCATTATGG	406bp	Sequences were obtained for 23 of 24 tested *Bartonella* species.	Oksi et al., 2013
	prAPT0245	GTCTCGTGGGCTCGGAGATGTGTATAAGAGACAGAATGGTGCCTCAGCACGTATAAG			
ssrA	ssrA_F	TCGTCGGCAGCGTCAGATGTGTATAAGAGACAGGCTATGGTAATAAATGGACAATG	262bp	Sequences were obtained for all tested *Bartonella* species.	This study
	ssrA_R	GTCTCGTGGGCTCGGAGATGTGTATAAGAGACAGGCTTCTGTTGCCAGGTG			Diaz et al., 2012
