# Supplementary figures and images for: Development of a quadruplex PCR amplicon next generation sequencing assay for detection and differentiation of Bartonella spp
Source: Front Microbiol. 2023 Dec 7;14:1243471. doi: 10.3389/fmicb.2023.1243471 (PMC10733521; doi:10.3389/fmicb.2023.1243471)

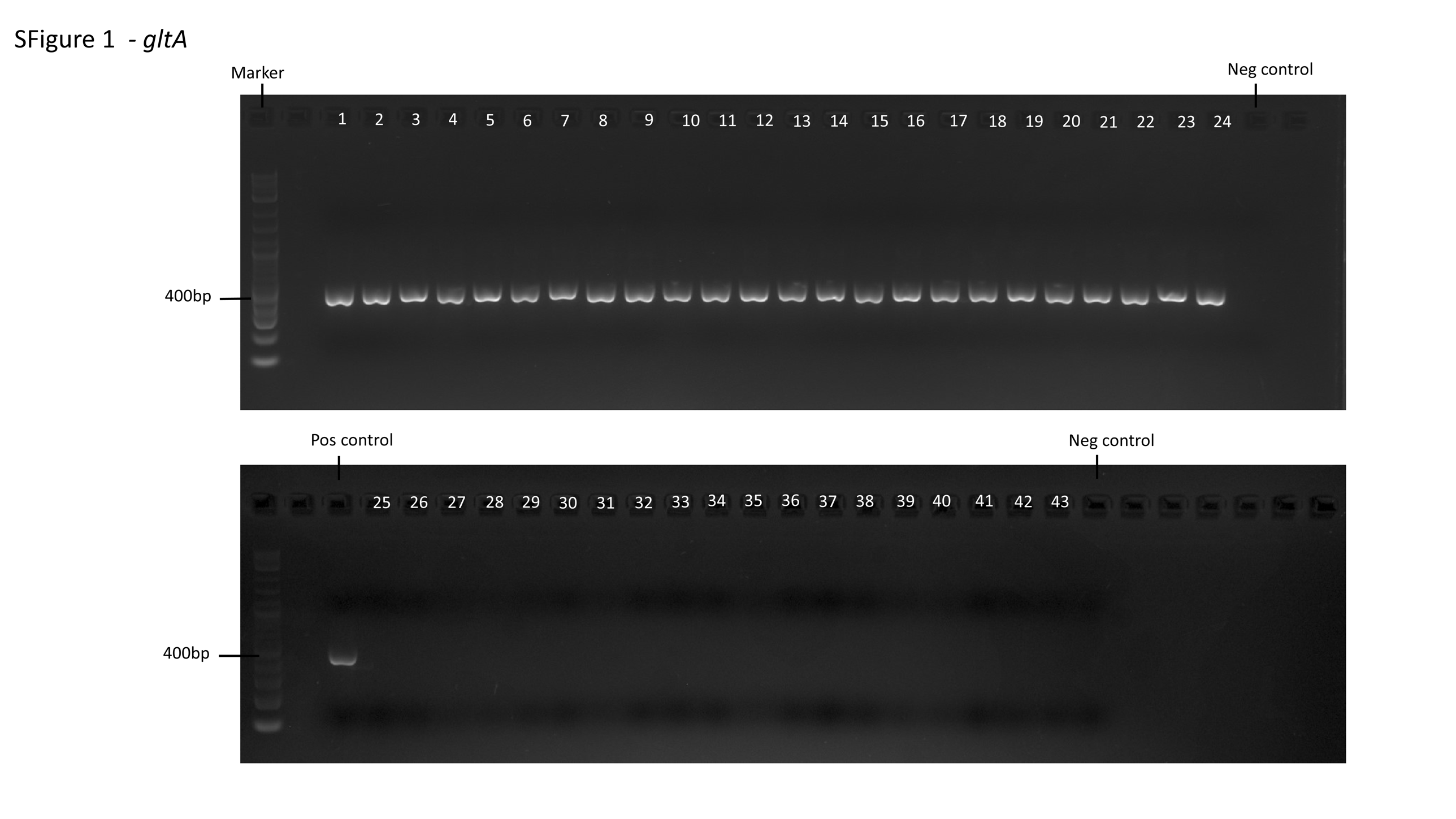

Supplement: Supplementary Figure S1 — Gel electrophoresis of gltA, Number 1–24 were the 24 Bartonella species (listed in Table 2) and number 25–43 were the 19 non-Bartonella species (listed in Section “DNA template of Bartonella species and non-Bartonella species) tested in the study. All Bartonella species was amplified and none of the non-Bartonella species was amplified. [file Image_1.TIF]

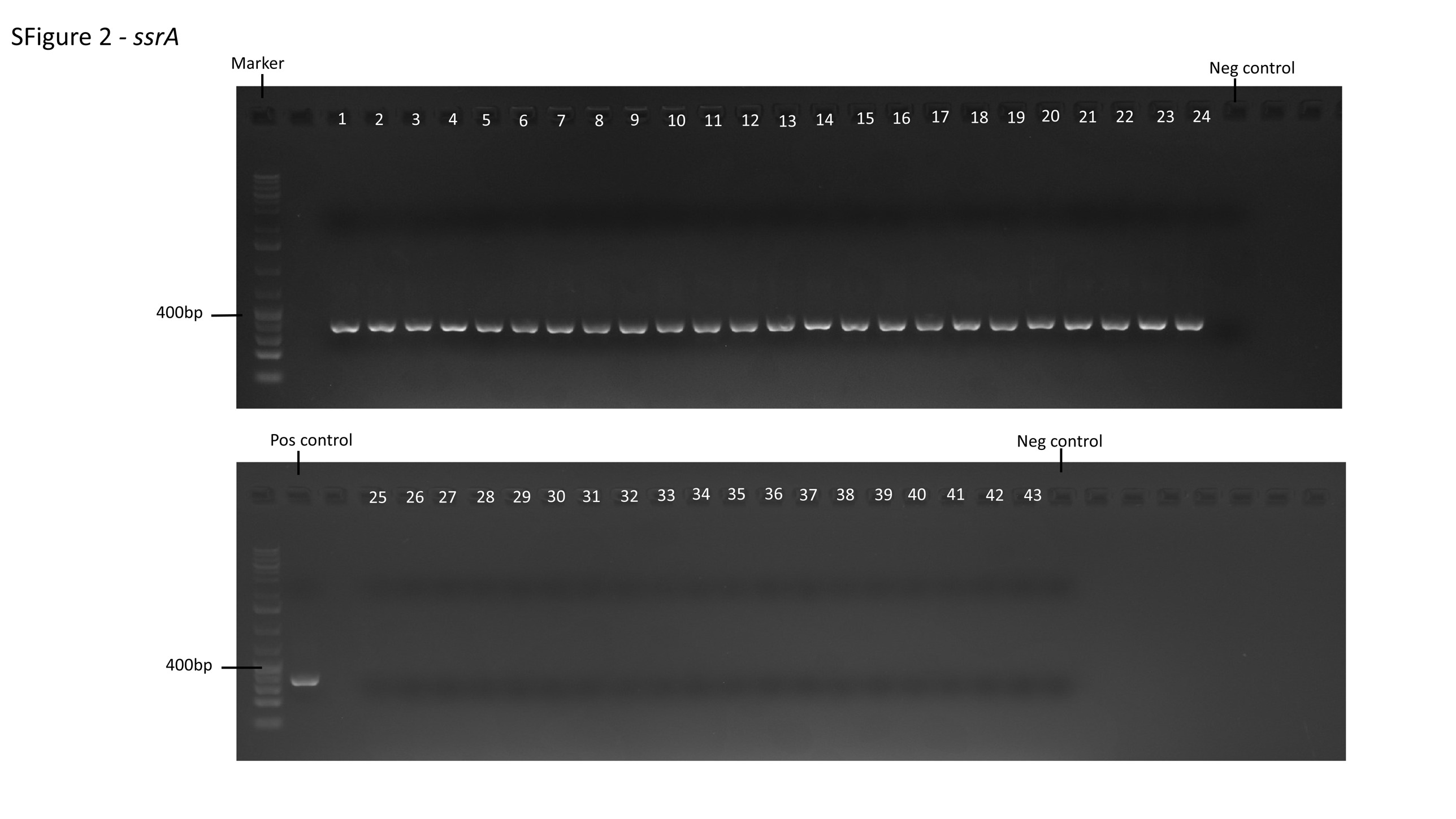

Supplement: Supplementary Figure S2 — Gel electrophoresis of ssrA. Number 1–24 were the 24 Bartonella species (listed in Table 2) and number 25–43 were the 19 non-Bartonella species (listed in Section “DNA template of Bartonella species and non-Bartonella species) tested in the study. All Bartonella species were amplified and none of the non-Bartonella species was amplified. [file Image_2.TIF]

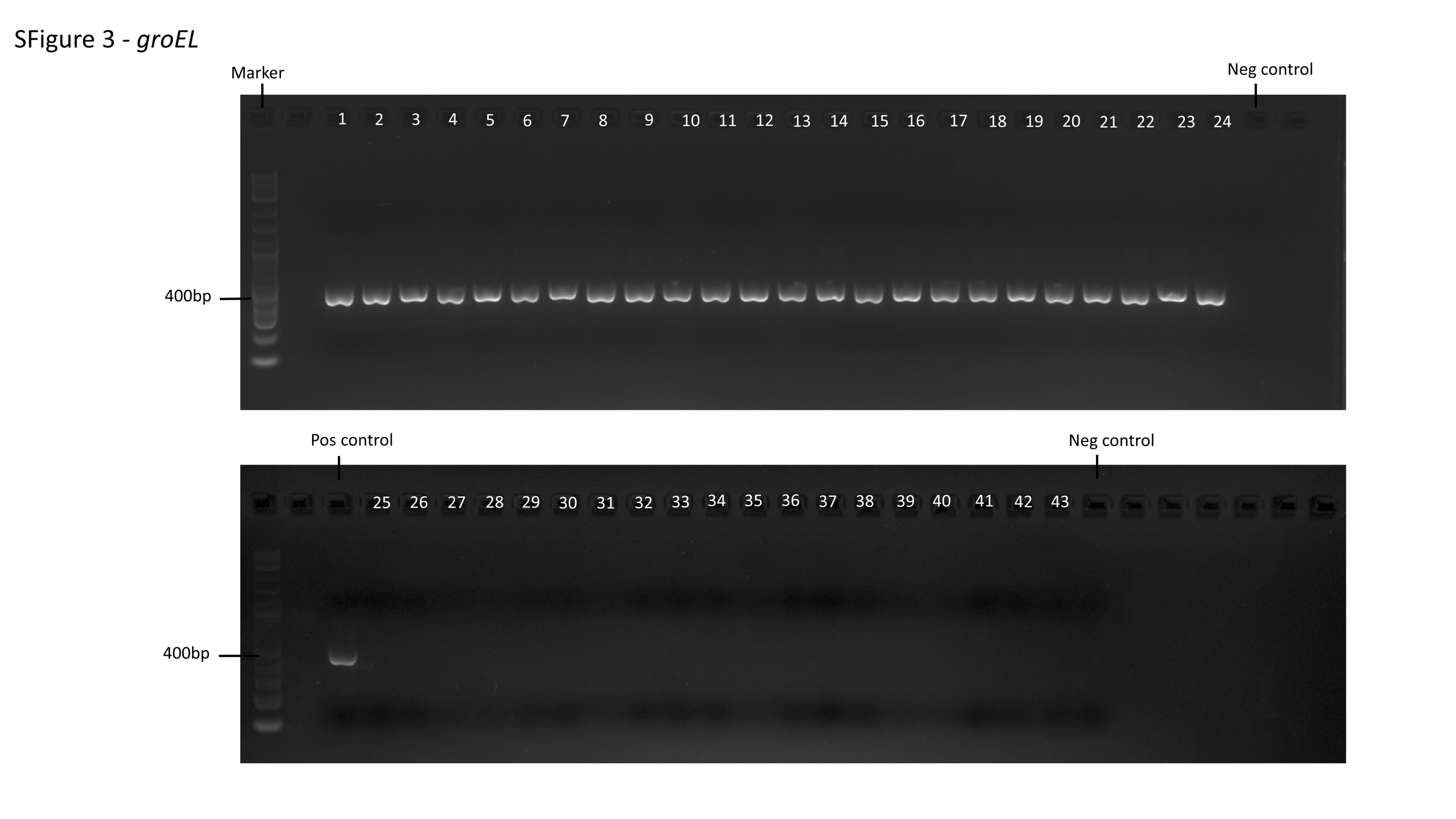

Supplement: Supplementary Figure S3 — Gel electrophoresis of groEL, Number 1–24 were the 24 Bartonella species (listed in Table 2) and number 25–43 were the 19 non-Bartonella species (listed in Section “DNA template of Bartonella species and non-Bartonella species) tested in the study. All Bartonella species was amplified and none of the non-Bartonella species was amplified. [file Image_3.TIF]

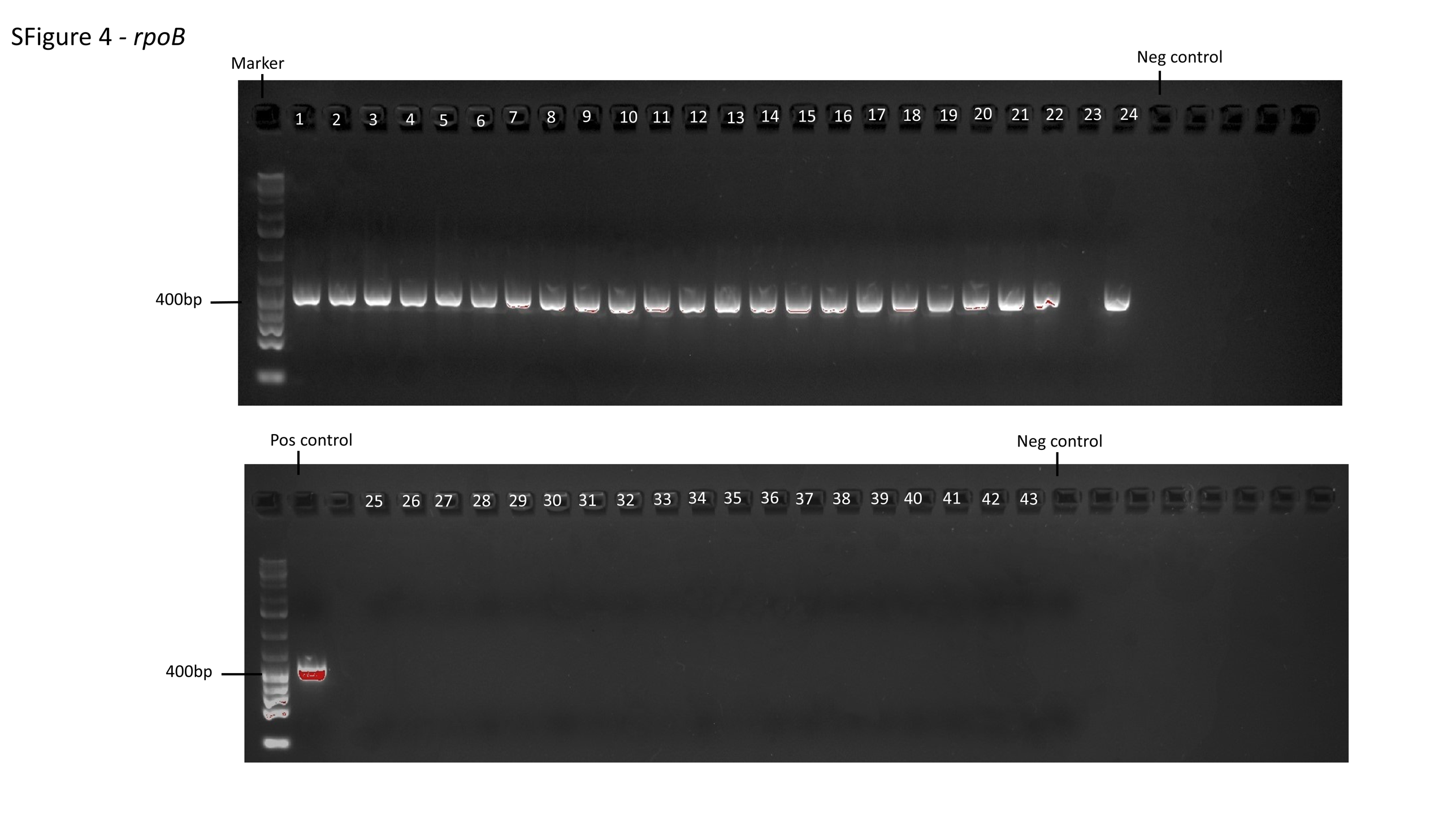

Supplement: Supplementary Figure S4 — Gel electrophoresis of rpoB, Number 1–24 were the 24 Bartonella species (listed in Table 2) and number 25–43 were the 19 non-Bartonella species (listed in Section “DNA template of Bartonella species and non-Bartonella species) tested in the study. All Bartonella species but B. clarridgeiae (#23) was amplified and none of the non-Bartonella species was amplified. [file Image_4.TIF]
